# Advanced Materials for Oral Application

**DOI:** 10.3390/ma15144749

**Published:** 2022-07-07

**Authors:** Laura-Cristina Rusu, Lavinia Cosmina Ardelean

**Affiliations:** 1 Department of Oral Pathology, Multidisciplinary Center for Research, Evaluation, Diagnosis and Therapies in Oral Medicine, “Victor Babes” University of Medicine and Pharmacy, 300041 Timisoara, Romania; laura.rusu@umft.ro; 2 Department of Technology of Materials and Devices in Dental Medicine, Multidisciplinary Center for Research, Evaluation, Diagnosis and Therapies in Oral Medicine, “Victor Babes” University of Medicine and Pharmacy, 300041 Timisoara, Romania

This Special Issue of *Materials* explores the wide variety of dental materials, which enables the dentists and dental technicians to select the most suitable therapeutic solution for each patient. The main goal of dental treatment concerns either the regeneration of diseased tissues or their replacement with prosthesis. Recent advances in this field enable the tailoring of dental materials to specific applications, resulting in progressive materials. The introduction of new aesthetic materials, digital devices, processing software, and manufacturing and prototyping tools has radically transformed the dental profession. Bioactive dental materials, which release specific ions, play an important role in the regenerative process, in preventive and restorative dentistry, and in endodontics or maxillofacial surgery, inducing cell differentiation and stimulation, promoting hard tissue formation, and exerting antimicrobial actions. Smart materials are able to react to pH changes and induce reparative processes in the oral environment [1,2].

This Special Issue aims to focus on the recent advances in this attractive field of research, which encourage a multidisciplinary approach to the subject.

This Special Issue contains a blend of 12 original research articles and 2 review papers from leading scientists across the world, with expertise in materials for dental application.

The reconstruction or repair of oral and maxillofacial functionalities and aesthetics is currently a priority for dental patients, as stated by Matichescu et al. in their review discussing “Advanced Biomaterials and Techniques for Oral Tissue Engineering and Regeneration” [3]. Tissue reconstruction is of utmost importance in oral and maxillofacial surgery, periodontics, orthodontics, endodontics, and even daily clinical practice. It involves a vast array of techniques ranging from the traditional use of tissue grafts to the most innovative regenerative procedures, such as tissue engineering, as well as a wide range of artificial and natural biomaterials and scaffolds, genes, oral stem cells, and growth factors. Due to the high rate of success, the future objective is achieving the regeneration of the entire tooth, which would replace classical dental implants and overcome their disadvantages [3].

Advanced platelet-rich fibrin, due to its concentrations of growth factors, is widely used to stimulate bone and soft tissue regeneration, currently being completely autologous and prepared without any anticoagulants or separators. The study by Sterczala et al. [4] aimed to determine whether combining advanced platelet-rich fibrin with autogenous fibroblasts results in increased release of components involved in the healing processes, such as fibroblast growth factor, vascular endothelial growth factor, transforming growth factor-beta 1 and 2, and collagen. The results have proven that advanced platelet-rich fibrin with autogenous human fibroblasts is a potential connective tissue substitute in the augmentation of keratinized gingiva, and it may considerably enhance the healing of surgical wounds [4].

Anorganic equine bone has been recently introduced as an alternative for bone grafting, used in oral surgery for volume preservation and for augmentation purposes. The aim of the study performed by Addis et al. [5] was to assess the physicochemical and structural properties of anorganic equine bone and its in vivo performance in mandibular bony defects. The study confirmed that its structural and physicochemical properties match the typical features of heat-treated xenogeneic bone substitutes, and its use as a grafting material yielded bone formation with no presence of inflammatory cell infiltrate [5].

Surgical reconstruction after radical surgery for malignant neoplasia was performed by Grigore et al. in the study “The Role of Biomaterials in Upper Digestive Tract Transoral Reconstruction” [6], aiming to provide novel biomaterials suitable for this purpose. Polydimethylsiloxane with silver nanoparticles was used for surgical reconstruction of the esophagus with templates, aiming to establish its antifungal properties. Following in vitro testing, the conclusion was that the insertion of bacteriostatic agents, such as silver nanoparticles, decreases the fatigue strength, increases flexibility, and offers an optimal local protection solution against fungal development [6].

Among the most recent and performant technologies used in prosthetic dentistry, computer-aided design and computer-aided manufacturing (CAD/CAM) enable subtractive or additive fabrication of various types of dental prostheses and appliances. The in vitro study by Nold et al., titled “Does Printing Orientation Matter? In-Vitro Fracture Strength of Temporary Fixed Dental Prostheses after a 1-Year Simulation in the Artificial Mouth” [7], aimed to compare the fracture resistance of milled and additive manufactured three-unit temporary fixed dental prostheses and bar-shaped specimens. The materials used were polymethylmethacrylate for subtractive manufacturing and a light-curing resin for additive manufacturing. Four different printing orientations were used for additive manufacturing. The subtractive manufactured bars and prostheses showed the highest strength in all experiments. The conclusion of the study was that fracture resistance is significantly affected by the manufacturing technique, the printing orientation, and the applied loading procedure [7].

Recent advances in CAD/CAM technologies have allowed the manufacturing of different types of materials for the CAD/CAM milling process. Contemporary CAD/CAM blocks are categorized into metal-based, ceramic-based, and resin-based. Polymer infiltrated ceramic network (PICN) composites are increasingly popular as CAD/CAM restorative materials because of their mechanical biocompatibility with human enamel. The study by Kawajiri et al. [8], “PICN Nanocomposite as Dental CAD/CAM Block Comparable to Human Tooth in Terms of Hardness and Flexural Modulus”, aimed to develop a novel PICN composite CAD/CAM block material comprising a silica skeleton and infiltrated UDMA-based resin. The proposed PICN nanocomposite represents a promising biocompatible material, as it exhibited a similar Vickers hardness to enamel and flexural modulus to dentin, in addition to excellent bond properties with resin cement [8].

Among the ceramic-based CAD/CAM blocks, lithium disilicate glass-ceramic represents a trending topic in prosthodontics due to its multifunctional use and translucency. The clinical performance of a fixed prosthetic restoration is influenced by the cement used to bond it to the dental structures. The aim of the study performed by Tribst et al. [9] was to evaluate the effect of different cement layer thicknesses on immediate and aged microtensile bond strength between lithium disilicate and dentin. The residual stress of polymerization shrinkage was assessed by applying the finite element method. The results showed that the cement layer thickness does not affect the immediate bond strength in lithium disilicate restorations. Thicker cement layers, due to the higher volume of material, induce higher residual stress; in the long term, the bond strength will be dampened. Therefore, to improve bond durability, a thinner cement layer is recommended [9].

Dental zirconia is another frequent choice of ceramic-based CAD/CAM block because of its versatility, combining high strength with acceptable aesthetics. Its lack of translucency has been overcome by the latest generations of 3Y-TZP zirconia, with improved properties and wider indications for both monolithic and veneered restorations. The study by Gasparik et al. [10] aimed to evaluate the masking ability of 1 mm thick monolithic and veneered zirconia crowns on different discolored substrates. Its importance lies in the fact that treating tooth discoloration is challenging, and a successful outcome relies on the material’s ability to hide the discolored substrate, as well as matching the color of neighboring teeth. The color measurements were performed using a non-contact dental spectrophotometer in the cervical, middle, and incisal portion of each crown. Despite the fact that the color coordinates of monolithic and veneered crowns were significantly different on all substrates, none of the 1 mm thick 3Y-TZP zirconia crowns showed sufficient masking ability on moderately or severely discolored substrates [10].

Despite the availability of digital impression procedures associated with the CAD/CAM techniques, conventional impressions are still widely used in daily dental practice. The impression material is usually handled by means of a dental tray; in certain cases, an adhesive is needed to provide a chemical adhesion between the material and the tray. The article by Paczkowski et al. [11] aimed to investigate the risk of cross-contamination when using dental tray adhesives with reusable brush systems. The risk of potential transmission for Staphylococcus aureus, Escherichia coli, Pseudomonas aeruginosa, Streptococcus oralis, and Candida albicans was determined for four dental tray adhesives with different disinfectant components. Isopropanol and ethyl acetate proved the most effective disinfectants, while hydrogen chloride and acetone were the least effective; however, all four tested adhesives showed sufficient bactericidal and fungicidal properties [11].

One dental field with great development within the recent time period has been endodontics. The ultimate goal of endodontic treatment is to obtain a three-dimensional, tight canal seal. Characterized by high biocompatibility, low cytotoxicity, and viscosity, tricalcium-silicate-based sealers have been considered to improving canal filling quality.

Nevertheless, as stated by Sfeir et al. in their review article “Calcium Silicate-Based Root Canal Sealers: A Narrative Review and Clinical Perspectives” [12], their dimensional stability is still questionable, with available studies showing contradictory results. Aiming to propose rational indications and help practitioners in selecting the appropriate sealer, the authors have shown that, compared to conventional sealers, calcium-silicate-based root canal sealers show good all-around performance, despite the significant differences between the different formulations. For this reason, their specificities must be considered by the practitioner when selecting the proper material for clinical usage, and slightly modified clinical endodontic protocols should be considered to match their specific behaviors [12].

From this perspective, two new modified calcium-silicate-based sealers, proposed with warm vertical gutta-percha obturation techniques, were evaluated by Eid et al. [13] to determine the impact of the warm vertical compaction on the dentinal tubule penetration. The in vitro study “Impact of Warm Vertical Compaction on the Sealing Ability of Calcium Silicate-Based Sealers: A Confocal Microscopic Evaluation” concluded that warm vertical compaction enhanced the penetration of calcium-silicate-based sealers into the dentinal tubules in comparison with the single cone technique [13].

Three-dimensional cleaning and shaping of the root canal system is the base for a proper obturation. Various nickel–titanium (NiTi) instruments have recently become available on the market. NiTi rotary files may undergo fatigue; novel manufacturing technologies such as reciprocating files or active cutting regions are aimed to overcome this deficiency. Alsofi et al. [14], in the article “Analysis of the Morpho-Geometrical Changes of the Root Canal System Produced by TF Adaptive vs. BioRace: A Micro-Computed Tomography Study”, aimed to evaluate and compare, using an ex vivo model, the shaping ability of adaptive reciprocation kinematics and continuous rotation instrumentation movement using TF Adaptive files and BioRace files, respectively. The conclusion was that both rotary systems produced canal preparations with adequate geometrical changes, but none of them could touch all the canal walls [14].

The materials used in restorative dentistry are also subject to continuous improvement, in terms of both physical properties and aesthetic appearance. Direct composite materials are the first choice when aesthetics is the primary goal. A wide choice of such materials is currently available, the match between the appearance of the restoration and the tooth structures depending not only on color but also on fundamental optical properties, such as translucency, opalescence, and fluorescence, indispensable for clinical shade-matching [15]. The article by Bardocz-Veres et al. aimed to investigate the fluorescence of nine different composite resins. They concluded that the fluorescence intensity of the assessed restorative materials shows significant differences compared to dental enamel, presenting lower or higher values. Further, all the tested composite resins showed decreased fluorescence values after six months [15].

Base materials are commonly used in dental practice to replace lost dentin and enable a uniform distribution of the load and tension, thus preventing tooth fracture. The in vitro study by Ciavoi et al. [16] aimed to compare the fracture resistance of teeth presenting medium-sized mesial–occlusal–distal cavities, restored with a light-cured composite resin, using different base materials: zinc polycarboxylate cement, glass ionomer cement, resin-modified glass ionomer cement, and flow composite. The study concluded that, in case of medium-sized mesial–occlusal–distal cavities, the base material with the highest fracture resistance is flow composite, followed by resin-modified glass ionomer cement, glass ionomer cement, and zinc polycarboxylate cement. In the authors’ opinion, one possible reason for this might be better compatibility with the final, light-cured restoration material [16].

In summary, this Special Issue of *Materials*, titled “Advanced Materials for Oral Application”, compiles a total of 14 cutting-edge research and extensive review articles demonstrating the great potential of novel, durable, and highly aesthetic dental materials in enhancing the quality of life for dental patients. The Special Issue also informs the readers of the current challenges and future directions in this domain. The Guest Editors would like to thank all contributing authors for the success of the Special Issue. This Special Issue would not have been of such quality without the constructive criticism of the Reviewers.
